# A Comparison of effect of preemptive versus postoperative use of ultrasound-guided bilateral transversus abdominis plane (TAP) block on pain relief after laparoscopic cholecystectomy

**DOI:** 10.1038/s41598-021-04552-6

**Published:** 2022-01-12

**Authors:** Poupak Rahimzadeh, Seyed Hamid Reza Faiz, Kaveh Latifi-Naibin, Mahzad Alimian

**Affiliations:** 1 Pain Research Center, Department of Anesthesiology and Pain Medicine, School of Medicine, Iran University of Medical Sciences, Tehran, Iran; 2 Minimally Invasive Surgery Research Center, Department of Anesthesiology and Pain Medicine, School of Medicine, Iran University of Medical Sciences, Tehran, Iran; 3 Department of Anesthesiology and Pain Medicine, School of Medicine, Iran University of Medical Sciences, Tehran, Iran

**Keywords:** Medical research, Clinical trial design

## Abstract

Nowadays, there are various methods to manage pain after laparoscopic cholecystectomy. The aim of this study was to compare the effectof preemptive versus postoperative use of ultrasound-guided transversus abdominis plane (USG-TAP) block on pain relief after laparoscopic cholecystectomy. In this single-blinded randomized clinical trial, the patients who were candidates for laparoscopic cholecystectomy were randomly divided into the two groups (n = 38 per group). In the preemptive group (PG) after the induction of anesthesia and in the postoperative group (POG) after the end of surgery and before the extubation, bilateral ultrasound-guided transversus abdominis plane (TAP) block was performed on patients using 20 cc of ropivacaine 0.25%. Both groups received patient controlled IV analgesia (PCIA) containing Acetaminophen (20 mg/ml) plus ketorolac (0.6 mg/ml) as a standard postoperative analgesia and meperidine 20 mg q 4 h PRN for rescue analgesia. Using the numerical rating scales (NSR), the patients’ pain intensity was assessed at time of arrival to the PACU and in 2th, 4th, 8th, 12th, 24th h. Primary outcome of interest is NSR at rest and coughing in the PACU and in 2th, 4th, 8th, 12th, 24th h. Secondary outcomes of interests were the time to first post-surgical rescue analgesic and level of patients’ pain control satisfaction in the first 24 h. The USG-TAP block significantly decreased pain score in the POG compared to the PG, and also the pain was relieved at rest especially in 8 and 12 h (*p* value ≤ 0.05) after the surgery. Pain score after coughing during recovery at 2, 8 and 12 h after the operation were significantly decreased. (*p* value ≤ 0.05) The patient satisfaction scores in the POG were significantly higher in all times. There was a statistically significant difference between the two groups in terms of rate of postoperative nausea and vomiting (PONV), indicating that patients in the POG had significantly lower incidences of the PONV compared tothe PG. The time to first analgesic request was significantly shorterin the POG, which was statistically significant (*p* value = 0.089). There was no statistically significant difference between the two groups in terms of consumption of analgesics. The postoperative TAP block could offer better postoperative analgesia than preepmtive TAP block.

## Introduction

Nowadays, Postoperative pain management is an important issue that has received a great deal of attention in the medical community. There are several methods for postoperative pain management, including the systemic analgesia (opioids and non-opioids) and the neuraxial anesthesia^1^. In addition to the side effects of opioid analgesics such as dizziness, respiratory depression, ileus, nausea, vomiting and itching, it is difficult to determine the appropriate dose of these opioids and achieve a steady state concentration, so the regional analgesic techniques have received much attention recently^2^. Peripheral nerve block is one type of these techniques and have received much attention recently because of their effective role in reducing postoperative pain and their better tolerance^3^. In order to improve quality of perioperative pain, the use of multimodal analgesia including the ultrasound-guided regional nerve along with non-steroidal anti-inflammatory drugs (NSAIDs) has received much attention recently^4^.

Post-laparoscopic cholecystectomy (LC) pain is multifactorial, and therefore multimodal analgesia has been suggested for its treatment. Visceral pain has been the primary source of postoperative pain in LC. Somatic or parietal pain in LC is less intense than visceral pain, owing to the small (1–4 cm) abdominal incisions of the trocar site and the limited damage to the abdominal wall but dealing with it could be as a part of multimodal analgesia which transversus abdominis plane (TAP) block can be an effective approach. Ultrasound-guided (USG) TAP block is one of the methods recommended for postoperative pain especially somatic pain control in the abdomen^5,6^. Several studies have demonstrated the efficacy of the TAP block in reducing postoperative pain scores and increasing the patient satisfaction^6^. The TAP block has been used to manage the preoperative pain, but few studies have been conducted on the timing of TAP block administration for the postoperative pain control.The aim of this study was to compare the effect the effect of preemptive versus postoperative use of the USG TAP block on pain relief after LC.

## Materials and methods

### Design and setting

This study was performed on the patients who were candidates for elective LC and referred to a university hospital,June 2020 to October 2020. The study protocol was approved by Ethical committee of Iran University (No: IR.IUMS.FMD.REC.1397.287, December 2018). The study was registered with the Iranian Clinical Trial registry at www.irct.ir: IRCT registration number: IRCT20120814010599N26; registration date: 31/05/2020 (https://en.irct.ir/trial/48012). All methods were carried out in accordance with relevant guidelines and regulations. All the ethical considerations of the latest version of Helsinki’s declaration were met throughout the study, and patients signed the written informed consent forms after receiving a complete explanation about the study.

### Eligibility criteria

The inclusion criteria were: (1) age range between 20 and 60 years, (2) ASA physical status of I-II according to American Society of Anesthesiologists classification, and (3) patients’ willingness to participate in the research. The exclusion criteria were: (1) Emergency cholecystectomy (2) history of opioid dependence or tolerance (2) conversion of laparoscopic cholecystectomy to open cholecystectomy (3) Unable to consent (4) history of allergy to ropivacaine (5) BMI > 35 (6) Coagulopathy (7) uncontrolled intraoperative bleeding. Eligible patients were randomized blindly into two groups: 38 patients in the preemptive group (PG) and 38 patients in the postoperative group (POG). For randomization, a simple randomization method is used, which is done using a table of random numbers. To use the number of random numbers, we first determine the reading path of the table numbers (for example, top, bottom, left or right). Then we assume certain numbers for each group (for example, even numbers for intervention A and odd numbers for intervention B). Then we touch on one of the numbers and move in one of the predetermined directions and record the numbers and assign them to different groups. Firstly, we did allocation and then blinding was done. The participants were not blinded to the allocation group. To prevent selection bias, allocation concealment was done in which the sequence of the group was unknown exactly before intervention.

### Intervention

With using this sequence, patients were assigned to PG or POG. Patients were anesthetized per research protocol: intravenous antibiotic prophylaxis according to the hospital's protocol, fentanyl 2 μg/kg, midazolam 0.12 mg/kg were used as premedication. Induction of anesthesia was performed by propofol 2 mg/kg and cisatracurium 0.2 mg/kg. Isoflurane1MAC (1.2%), cisatracurium 2 mg every 30 min. All patients were monitored by non-invasive blood pressure (NIBP), pulse oximetry, electrocardiography (ECG), capnography (ETCO2), and BIS. ETCO2 was kept 30 to 35 mmHg and BIS 40 to 60. There were four trocars entry points (measured 1–5 cm) were used- one at the umbilicus and three in the right upper abdominal quadrant. Intraperitoneal Co2 was insufflated to the intrabdominal with pressure of less than 15 mm Hg. Ondansetron 4 mg and paracetamol 1 gr were given in the last 20 min intravenous. After evacuating the pneumoperitoneum, patients were reversed by neostigmine and atropine and extubated. Fluid and electrolyte were managed based on the standard of care.

In both group, standardized monitoring (ECG, pulse oximetry, and NIBP) were applied on arrival to the post anesthesia care unit (PACU). Patient controlled intravenous analgesia (PCIA) containing 20 mg/ml of Acetaminophen and 0.6 mg/ml of Ketorolac with a bolus bottom (2 ml every 15 min) was started for each patient upon arrival to the PACU.

In the PG after the induction of anesthesia and in the POG after the end of surgery and before the extubation, the USG TAP block (Fuji Film Sonosite S-Nerve, Bothel, WA, USA) was performed on patients rested in a supine position using linear probe (5–13 MHZ). To perform the TAP block, the ultrasound probe was placed longitudinally on the midaxillary line near umbilicus, and the transversus abdominis and internal oblique muscles were scanned and observed. The needle (22-gauge 90 mm disposable spinal needle) was inserted in plane and after placing the needle-tip into the fascia between transversus abdominis and internal oblique muscles, 20 ml of ropivacaine 0.25% was injected bilaterally. It was all done by one Anesthetist which was expert in that area and was not in charge of collecting the data.

### Primary outcome

Pain intensity assessed by numerical rating scale (NRS) during rest and requested deep coughing, providing a range scores from 0 (no pain) to 10 (severe pain), by a blind assessor at six different times including: T0: on arrival to the PACU, T2:2th h, T4:4th h, T8:8th h, T12:12th h and T24:24th h.

### Data collection

Patients were assessed regarding pain intensity using NRS during rest and requested deep coughing, providing a range scores from 0 (no pain) to 10 (severe pain),by a blind assessor at six different times including:T0:on arrival to the PACU, T2:2th h, T4:4th h, T8:8th h, T12:12th h and T24:24th h. After using PCIA, if the patient had NRS > 3, Meperidine 20 mg was also prescribed as rescue analgesia at every time during the postoperative period for 24 h. Same blind assessor evaluated the patients satisfaction with a satisfaction score (0: poor, 1: moderate, 2:good, 3:very good, 4:excellent) were also reported at the end of the 24 h. Indeed, outcome assessor and the person analyzing the information were unaware of the study groups.

### Statistics analysis

A sample size of 76 was determined according to a study by Suseelaet al^7^, using the mean difference formula for two independent groups (Group I: 0.3 ± 0.56; Group II: 0.03 ± 0.16) with power of 80% (Z_1-β_ = 0.84), the significance level/alpha of 0.05 (Z_1-α/2_ = 1.96) and d (the minimum difference between the groups under study that would be of biological relevance) of 0.27 as following:

$${\text{n}} = \frac{{\left( {{\text{Z}}_{(1 - \upalpha /2)} + {\text{Z}}_{(1 - \upbeta /2)} } \right)^{2} \left( {{\text{sd}}_{1}^{2} + {\text{sd}}_{2}^{2} } \right)}}{{{\text{d}}^{2} }}$$

The statistical analysis for comparing the effectiveness of two methods was conducted as follows: for comparison of gender, medical history, vomiting, and the request for narcotics between two groups (post-op and preemptive) the chi-square tests were applied. For comparison of age, height, weight, and BMI and dosage of narcotics using Mann–Whitney U test were conducted. Comparison of NRS scores and satisfaction on 0, 2, 4, 8, 12, 24 h were separately conducted using Freidman test between two groups. Graphs show the mean and SEM of NRS and satisfaction scores. All analysis was done in Matlab 2018b.

### Ethics approval

The study protocol was approved by the Ethics Committee of Iran University of Medical Sciences, Tehran, Iran (IR.IUMS.FMD.REC.1397.287). All the ethical considerations of the latest version of Helsinki’s declaration were met throughout the study, and patients signed the written informed consent forms after receiving a complete explanation about the study. The study was registered with the Iranian Clinical Trial registry at www.irct.ir: IRCT registration number: IRCT20120814010599N26; Registration date: 31/05/2020; https://en.irct.ir/trial/48012.

### Informed consent

Informed consent was obtained from the patients.

## Results

**Figure 1:**
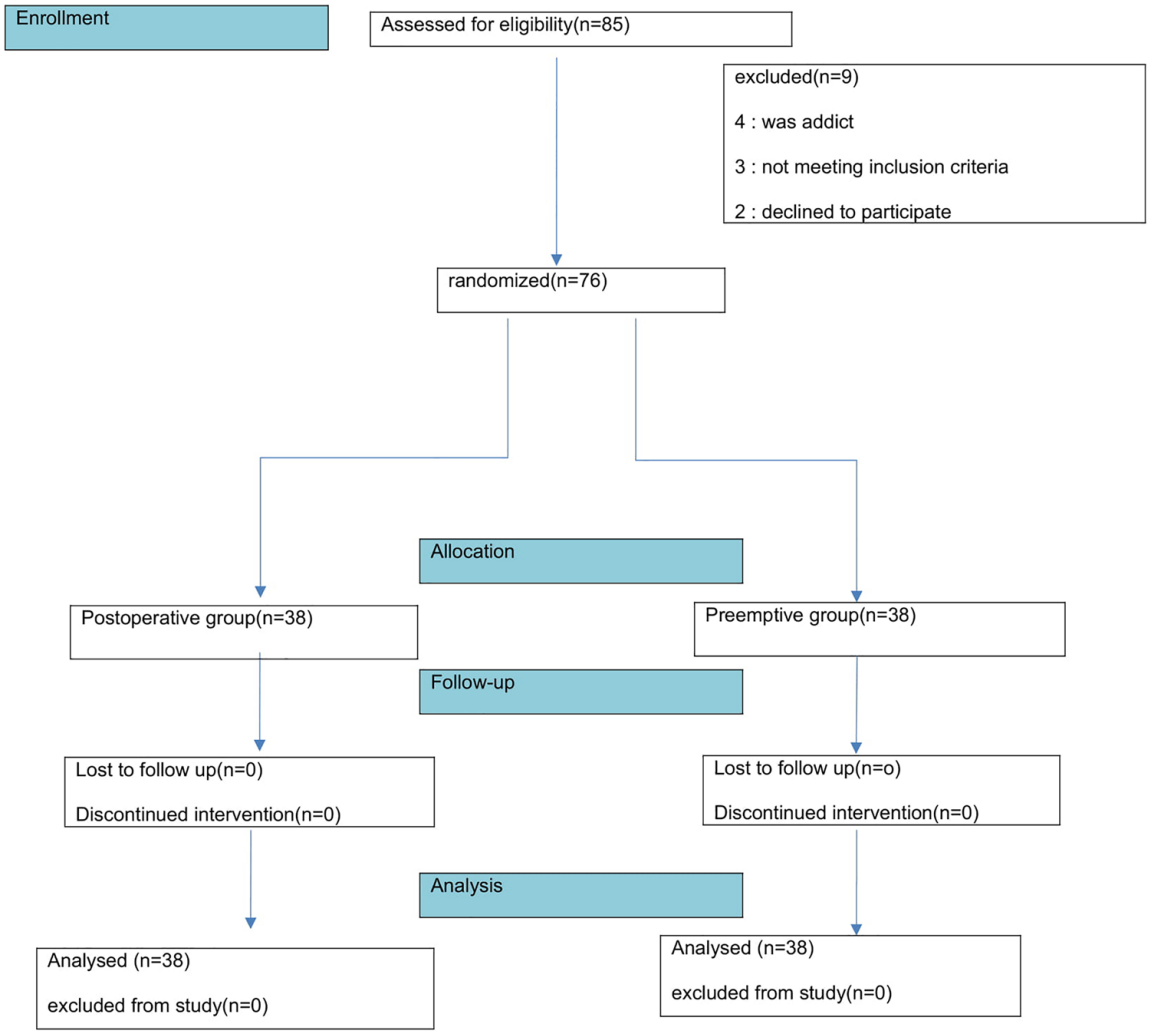
Flow chart of the study.

A total of 85 patients who were candidates for elective LC were enrolled in this study. Nine patients (4 for substance abuse, 2 refused to participate in the study, 3 for having no study eligibility criteria) were excluded from the study. As shown in consort flow diagram (Fig. 1). Finally, a total of 76 patients were included in both groups (n = 38 per group).

There were no statistically significant differences between the two groups with respect to demographic characteristics including age, weight, BMI, height and operation time (Table 1). Need to first opioid in the two groups compared. POG showed better analgesia in this regard, which had later analgesia request and the difference between them was significant (*p* = 0.089), there was no statistically difference between the two groups in terms of Postoperative Meperidine Consumption (*p* = 0.518) (Table 1). There was no complication in the groups but patients in the POG had significantly lower incidences of the PONV compared to the PG. (Table 2).

**Table 1:** Demographic characteristics and opioid request of the two group patients.

Variable		Mean	SD	Mean rank	*p* value
Height	PG	169.72	6.39	30.98	0.143
	POG	167.961	5.23	24.67	
Age	PG	45.000	10.87	29.71	0.743
	POG	44.46	8.30	28.27	
Weight	PG	71.34	9.97	27.74	0.718
	POG	75.44	17.82	29.31	
BMI	PG	24.69	2.56	25.33	0.191
	POG	27.11	7.58	30.98	
First opioid Request (hr.)	PG	2.22	1.20	5.67	0.089
	POG	5.80	2.68	10.80	
Operation Time (min)	PG POG	110.11 106.32	32.9 26.2	47.6 42.9	0.67
Meperidine consumption (mg)	PG POG	32.11 34.21	2.67 3.64	20.23 21.34	0.518

**Table 2:** Frequency of complication, gender and medical history between the two groups.

Variable		PG	POG	X^2^ statistic	*p* value
Vomiting	Yes	13	5	5.294	0.011
	No	16	24		
Gender	Male	16	19	0.650	0.592
	Female	13	10		
Medical history	Yes	8	7	0.764	> 0.999
	No	21	22		

**Figure 2:**
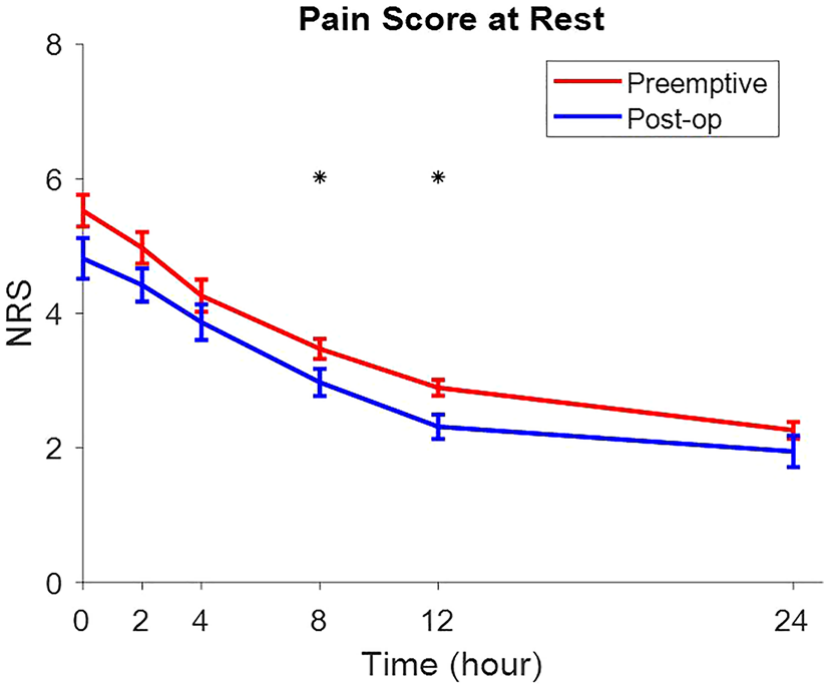
NRS scores at rest were decreasedin both groups over time, which were statistically significant in 8 and 12 h after the operation (*p* value = 0.04 and = 0.00 respectively) (Fig. 2).

NRS scores at rest were decreasedin both groups over time, which were statistically significant in 8 and 12 h after the operation (*p* value = 0.04 and = 0.00 respectively) (Fig. 2).

**Figure 3:**
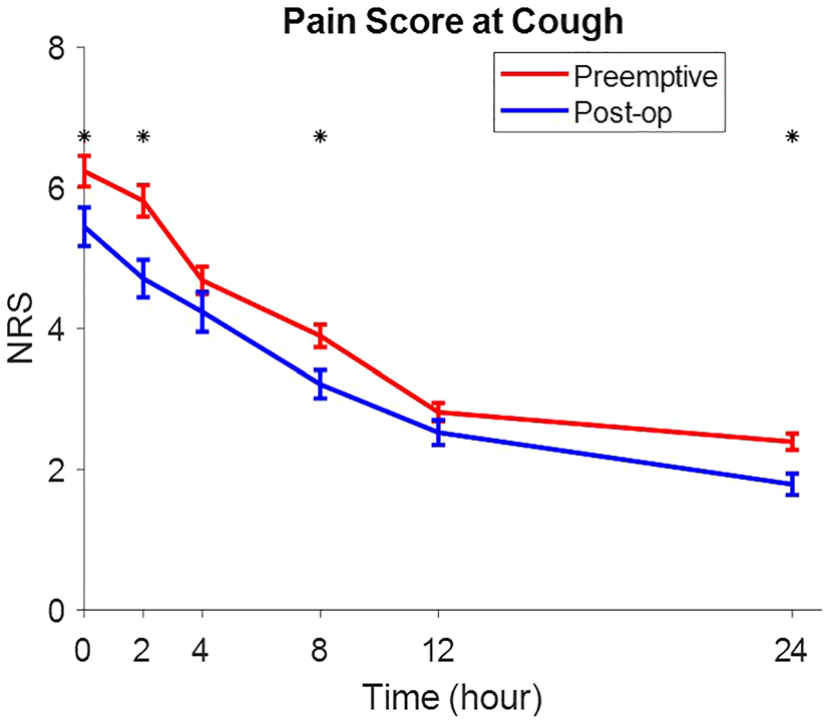
NRS at cough were decreased in both groups over time, which were statistically significant during the recovery at 2, 8, 12 h after the operation (*p* value = 0.027, *p* value = 0.002, *p* value = 0.009, and *p* value = 0.002, respectively).

NRS at cough were decreased in both groups over time, which were statistically significant during the recovery at 2, 8, 12 h after the operation (*p* value = 0.027, *p* value = 0.002, *p* value = 0.009, and *p* value = 0.002, respectively) (Fig. 3).

**Figure 4:**
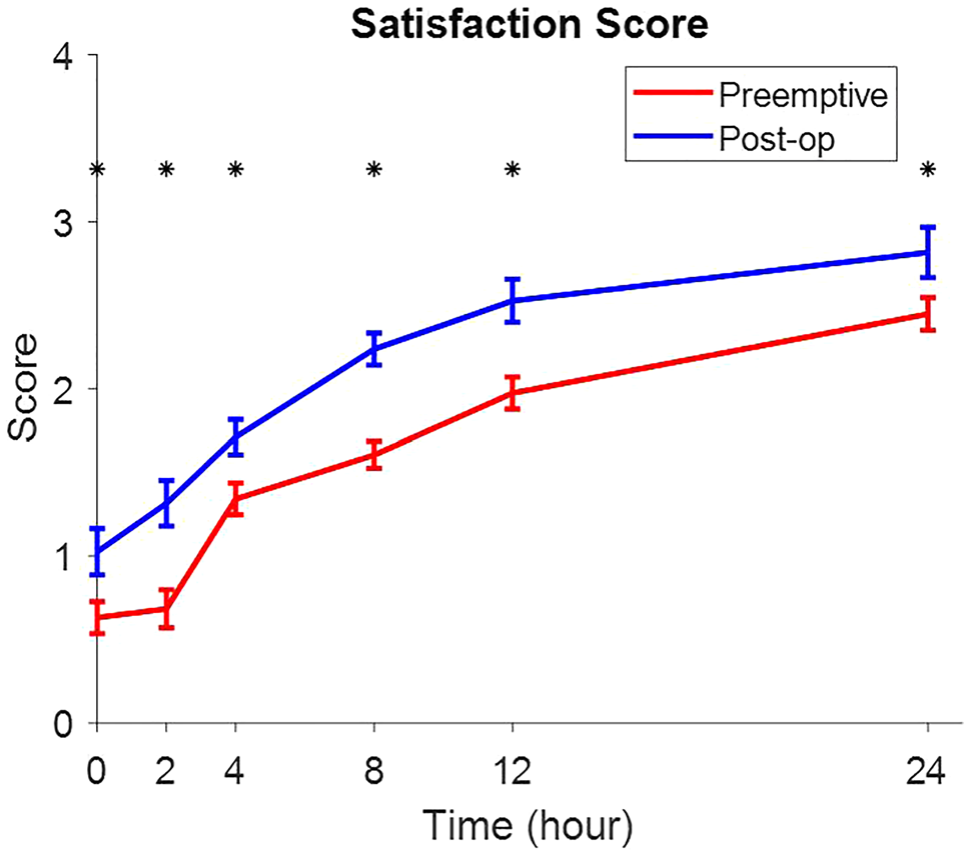
Patient satisfaction scores were significantly increased in both groups in all times (Fig. 4).

Patient satisfaction scores were significantly increased in both groups in all times (Fig. 4). No complications after block was reported.

## Discussion

Several studies have shown that the USG TAP blockas a part of multimodal analgesia for postoperative pain management, results in better analgesia, higher satisfaction, and greater opioid sparing effects^8–10^. Given that in patients undergoing LCsurgery, the maxim level of pain was in the first 24 h after surgery that was in trocar sites^8^, determining the optimal time to perform TAP blockeven before or at the end of surgery to increase blockefficiency is of clinical importance.However, few studies have been conducted on the comparison of analgesic effect between preepmtive and postoperative USG TAP blocks on pain relief after LC. In the current study, USG TAP block was performedin PG after the induction of anesthesia and in the POG at the end of surgery and before the extubation. Our results demonstrated that the USG TAP block decreased NRS scores both at rest and during coughing in the PG compared to the POG up to 24 h after the operation, especially in the first 8 h after the operation, which were not consistent with previous studies. In the Rashid study^11^ no significant difference was observed between the two groups in term of the amount of analgesia when performing dual TAP block and port site local anesthetic infiltration. Richard Kalu^12^ compared the effect of preoperative versus postoperative use of TAP on postoperative opioid use and the results showed that TAP block reduced the postoperative pain in both groups, but there was no significant difference between the groups with respect to the opioid use which was similar in our results. Our findings were consistent with those of the study of Tikuisis et al.^13^ demonstrating that TAP block reduced the pain scores both at rest in 2, 4, and 12 h after the operation and during coughing 2 and 4 h after the operation. A regional block of the abdominal wall, when used as a part of multimodal analgesia, can significantly relieve postoperative pain. Theblind TAP block technique based on an anatomical landmark can cause the complications and even injury to the abdominal viscera such as liver injury and intestinal puncture^14^. In the present study, there were no complications attributable to the TAP block because we performed real-time ultrasound guidance for TAP block in the patients. There was also no vital anatomical structure in this area. Our results showed thatthe USG-TAP block reduced the use of pethidinein the POG as compared with the PG, which in turn decreased the side effects of opioid analgesics such as dizziness, nausea, vomiting, itching, etc. In the present study, there were no complications attributable to block, indicating that by selecting the appropriate local anesthetic dose for TAP block, it could be used safely to reduce postoperative pain.Some studiesalso reported a beneficial effect of USG-TAP block on pain 48 h after surgery, which may be due to the relatively small presence of vascular structures in the neurovascular plane of the abdominal wall, as these structures play an important role in the drug clearance. Accordingly, systemic toxicity from local anesthetics has decreased significantly usingthe USG-TAPblock^15,16^. Patient satisfaction scores at all times during the first 24 h after surgery was statistically significant. This result was consistent with the study of Huang et al.^17^ comparinganalgesic efficacy of trocar sites local anesthetic infiltration with and without transversus abdominis plane block after laparoscopic hysterectomy and the results showed that patient satisfaction score was significantly higher in the TAP group. In the current study, the USG-TAP block reduced the opioid use and its side effects, especially PONV, but this reduction was statistically significant in the POG. Our Study showed postoperative TAP block appeared to be more efficacious than preoperative one in longer analgesia and lower pain scores.

### Limitations

However, this studyhas some limitations. One of the limitations of the study was the lack of evaluation of the effects of local anestheticswith the different doses and concentrations as some studies have shown that the use of higher doses relieves the pain and reducesopioid use in the parents^18^. Another limitation of our study was the lack of Sensory assessment of theTAP block because the appropriate sensory level caused by the USG-TAP block is important for its efficiency and the evaluation of the patient's pain^19,20^. Another limitation of the study is the small sample size and single center study.

## Conclusion

The USG-TAP block, as a part of multimodal analgesia for postoperative pain management, resulted in better analgesia and higher satisfaction in the POG compared to the PG. The opioid sparing effect of the TAP block reduced the side effects of opioid use, including nausea and vomiting. TAP block could be considered as an integral part of multimodal analgesic strategy that is an inexpensive, simple and easily performed procedure.
